# Gate-Level Circuit Partitioning Algorithm Based on Clustering and an Improved Genetic Algorithm

**DOI:** 10.3390/e25040597

**Published:** 2023-03-31

**Authors:** Rui Cheng, Lin-Zi Yin, Zhao-Hui Jiang, Xue-Mei Xu

**Affiliations:** 1 School of Physics and Electronics, Central South University, Changsha 410083, China; 2 School of Automation, Central South University, Changsha 410083, China

**Keywords:** circuit partitioning, clustering algorithm, genetic algorithm, betweenness centrality

## Abstract

Gate-level circuit partitioning is an important development trend for improving the efficiency of simulation in EDA software. In this paper, a gate-level circuit partitioning algorithm, based on clustering and an improved genetic algorithm, is proposed for the gate-level simulation task. First, a clustering algorithm based on betweenness centrality is proposed to quickly identify clusters in the original circuit and achieve the circuit coarse. Next, a constraint-based genetic algorithm is proposed which provides absolute and probabilistic genetic strategies for clustered circuits and other circuits, respectively. This new genetic strategy guarantees the integrity of clusters and is effective for realizing the fine partitioning of gate-level circuits. The experimental results using 12 ISCAS ‘89 and ISCAS ‘85 benchmark circuits show that the proposed algorithm is 5% better than Metis, 80% better than KL, and 61% better than traditional genetic algorithms for finding the minimum number of connections between subsets.

## 1. Introduction

Gate-level circuit partitioning is a very important phase during EDA simulation [1]. It divides large-scale circuits into similar-sized subsets, with a minimum number of connections between subsets. The quality of circuit partitioning directly affects the sequence simulation [2,3,4]. With the rapid increase in chip integration, gate-level circuit partitioning algorithms are attracting expanding attention from the industry and scholars, becoming an essential part of new generation EDA simulation software. There are two key indicators to evaluate a circuit partitioning algorithm: the minimum number of connections and load balancing. Early circuit partitioning algorithms mainly include KL [5,6,7] and FM [8,9]. With the development of machine learning theory, some heuristic algorithms, such as the genetic algorithm [10,11,12,13,14], the particle swarm optimization algorithm [15,16], the bird flock algorithm [17], etc., have also emerged. In order to further improve the calculation speed, multi-level partition algorithms [18,19,20], such as Metis [21] and /hMetis [22], etc., have received extensive attention in recent years. Kumar [23] proposed a streaming Metis partition to alleviate the computational resource constraints when dealing with large graphs. He applied the traditional multi-level graph partition strategy to divide ultra-large-scale circuits [24].

In general, multi-level partition algorithms include two phases: the coarse partition phase and the fine partition phase. The former identifies clusters in the original circuit and achieves circuit coarsening. The latter classifies the other nodes for the minimum number of connections and load balancing. Although there are many algorithms to identify the clusters of a circuit, the clustering algorithm is the most popular and effective to identify similar nodes at the same time [25]. Clustering plays an important role in many scientific fields [26], including earth sciences [27,28], biology [29,30,31], economics [32], community detection [33], etc. The nodes identified by clustering algorithms are called clusters, which is very important for rapidly realizing the coarse partitioning of a circuit. However, the traditional clustering algorithms suffer from some disadvantages when they are applied to gate-level circuit coarse partition, including the random search starting node and how to determine the optimal cluster size, etc. Thus, it is necessary to design an efficient clustering algorithm based on the gate-level circuit features.

Moreover, these traditional multi-level partition algorithms, such as Metis, would break the related clusters of the original circuits. This means that their fine partition modules would contradict the conclusions of the coarse partition modules, to some extent. Furthermore, these traditional algorithms often prioritize load balancing and treat the minimum number of connections as a minor condition. For example, in the fine partition phase, the traditional Metis algorithms are often optimized by the peer-to-peer exchange of the elements in different subsets, which strictly guarantees load balancing, but may lead to an increase in the number of connections. For gate-level circuit partitioning, the number of connections between subsets, called cutsize, is key to reduce the waiting delay when simulating different partition subsets. Thus, it is necessary to design a new fine partition algorithm which not only ensures the integrity of clusters to achieve the compatibility with the coarse partition algorithm, but also takes the minimum cutsize as the most important optimization target. 

In order to resolve the above disadvantages, a new gate-level circuit partitioning algorithm based on clustering and an improved genetic algorithm is proposed. The proposed algorithm adopts a two-level partition structure. In the coarse partition phase, the notions of degree and betweenness centrality [34,35,36,37] of the graph theory are applied to optimize the search starting node and identify the boundary of a cluster, respectively. They are effective to improve the computational efficiency of clustering algorithms and determine the related clusters. In the fine partition phase, a constraint-based genetic algorithm is proposed which adopts the absolute genetic strategy for nodes in clusters and the probabilistic genetic strategy for other nodes. This new genetic strategy can effectively realize the seamless connection with the coarse partition, greatly reduce the search space, and improve the convergence speed. In addition, the proposed genetic algorithm takes the minimum cutsize as the optimization objective of the fitness calculation, and can obtain a relatively better partition scheme, with a minimum cutsize.

The main contributions of this paper include the following: (1) a new gate-level circuit partitioning algorithm is proposed; (2) a clustering algorithm based on betweenness centrality is proposed which can identify and preserve clusters to realize the coarse partition of a gate-level circuit; and (3) a constraint-based genetic algorithm is proposed, combining absolute genetic strategy and probabilistic genetic strategy, which realizes a seamless connection with coarse partition and is effective to obtain better partition results.

## 2. Preliminary Knowledge

A gate-level circuit can be described as an undirected graph *G*(*V*, *E*), where $V=\left\{v_{1},v_{2},\cdots ,v_{n}\right\}$ is a set of nodes that represents the set of electronic components. $E=\left\{e_{1},e_{2},\cdots ,e_{m}\right\}$ corresponds to the set of graph edges that represents the set of connections between electronic components. The number of edges connected to node v, called the degree of node v, is denoted as $d_{G}\left(v\right)$ or d(v).

Given an integrated circuit graph *G*(*V*, *E*), if all the nodes are divided into *k* two-two disjoint subsets $\left\{V_{1},V_{2},\cdots ,V_{k}\right\}$ and $V_{1}\;\cup \;V_{2}\;\cup \;\cdots \;\cup \;V_{k}=V$, then the union of subsets is referred to as a *k*-way partition of graph *G*. Considering a given balance factor *β*, the *k*-way partition is considered to be load balancing if it satisfies the following:

For any $V_{p}\in V$, 1 < = *p* < = *k*, $\left|\;V_{p}\;\right|$ represents cardinality, that is, the number of nodes in $V_{p}$, which needs to satisfy Equation (1):

(1) $$(1/k)\cdot (1\;-\;\beta )\sum_{i=1}^{k}\left|V_{i}\;\right|\;\leq \;\left|\;V_{p}\;\right|\leq \;\left(1/k\right)\;\left(1+\beta \right)\;\sum_{i=1}^{k}\left|V_{i}\right|$$

**Figure 1:**
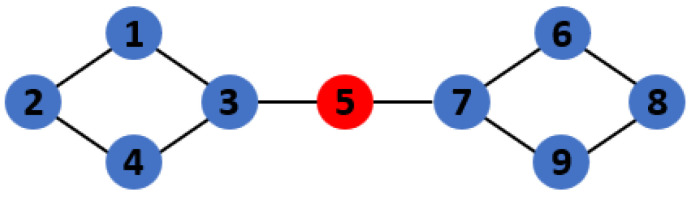
Schematic diagram of betweenness centrality. The betweenness centrality of nodes 1–9 is {0.107, 0.018, 0.554, 0.107, 0.571, 0.107, 0.554, 0.018, 0.107}, and node 5 has the highest betweenness. That is, node 5 connects two clusters, {1, 2, 3, 4} and {6, 7, 8, 9}.

Betweenness centrality provides a general standard for the measurement of graph centrality. For any node $v_{p}\in V$, the betweenness centrality $c\left(v_{p}\right)$ represents the probability sum of the shortest path through node $v_{p}$:

(2) $$c\left(v_{p}\right)=\sum_{v_{i},v_{j}\in V}\frac{\sigma \left(v_{i},v_{j}|v_{p}\right)}{\sigma \left(v_{i},v_{j}\right)};p\;\neq \;i\;\neq \;j$$

where $\sigma \left(v_{i},v_{j}\right)$ is the number of shortest paths between any node $v_{i}$ and $v_{j}$, and $\sigma \left(v_{i},v_{j}|v_{p}\right)$ is the number of shortest paths that contain node $v_{p}$. Nodes with high betweenness centrality play the role of a broker, or gatekeeper, to connect the nodes and sub-groups [36]. In other words, it is a “bridge connection” of circuit clusters. A simple example is shown in Figure 1. 

For ease of expression, we define the concepts of the high betweenness node set and the non-high betweenness maximum degree node to identify the boundary and starting node of the clustering algorithms, as follows:

**Definition 1.**  *Given a gate-level circuit G = (V, E), a node set Y ⊆ V is called the high betweenness node set, if it satisfies the following two conditions*:

1. |*Y*| = *k − 1*;
2. *For* ∀ $v^{\prime }\in V\backslash Y$, ∀ v∈Y, *it has c*(*v*) ≥ *c*(*v′*).

In detail, the high betweenness node set Y contains *k* − 1 nodes with the highest betweenness centrality.

**Definition 2.**  *Given a gate-level circuit G = (V, E), and a high betweenness node set Y, node *$v_{max}$ *is called a non-high betweenness maximum degree node if it satisfies the following two conditions:*

1. $v_{max}\notin Y$;
2. ∀v∈V\Y, $d\left(v_{max}\right)\;$≥ d(v).

The node $v_{max}$ is mainly used as the starting node for each round of clustering, and it is necessary to ensure that this node is not in the high betweenness node set *Y*.

## 3. Gate-Level Circuit Partitioning Algorithm Based on Cluster and an Improved Genetic Algorithm

**Figure 2:**
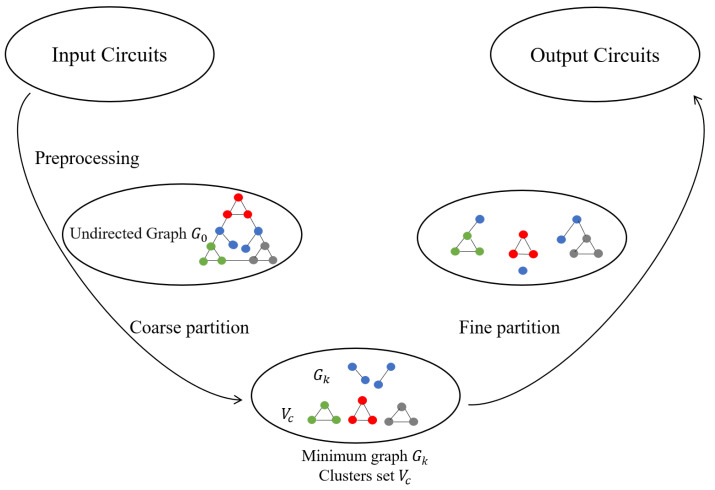
Schematic diagram of the three-way partition of the circuit under a two-level partition structure. During the preprocessing phase, all the electronic components in the circuit are unified, and the undirected graph $G_{0}$ is outputted. During the coarse partition phase, the clustering algorithm reduces the scale of $G_{0}$ and outputs cluster set *Vc* and minimum graph $G_{k}$. During the fine partition phase, the improved genetic algorithm assigns nodes in $G_{k}$ to each cluster and outputs the circuits with the minimum cutsize.

The goal of circuit partitioning is to divide large-scale circuits into similar-sized subsets, with a minimum cutsize. First, a real gate-level circuit stored in the netlist file was modeled into an undirected graph through preprocessing. Next, a two-level partition structure was adopted to obtain the smallest cutsize and guarantee a certain load balancing. In detail, we proposed a clustering algorithm based on betweenness centrality to realize coarse partitioning of the original gate-level circuit and to guarantee a certain load balancing. On this basis, a constraint-based genetic algorithm is proposed to realize a fine partition for the minimum cutsize. The various phases of the three-way partition of the circuit under a two-level partition structure are shown in Figure 2.

The proposed algorithm, that is, the gate-level circuit partitioning algorithm based on the clusters and the improved genetic algorithm, works only for undirected graphs, and is not directly applicable to circuits. The algorithm mainly includes the following two parts:

1. The clustering algorithm based on betweenness centrality. It applies BFS (breadth-first search) to identify the clusters in a graph and realize the coarse partition. These clusters can ensure a certain load balancing and greatly reduce the scale of the graph; that is, the solution space of the subsequent fine partitioning algorithm is reduced.
2. The constraint-based genetic algorithm. It adopts the absolute genetic strategy for nodes in clusters and the probabilistic genetic strategy for other nodes, so as to achieve the rapid convergence of the algorithm and to match with the coarse partition. The genetic algorithm takes the minimum cutsize as the optimal goal and outputs the best partition scheme.

### 3.1. Gate-Level Circuit Modeling and Preprocessing

A gate-level circuit is typically stored in a text file containing instantiated logical gates and port-map-based connections. Circuit partitioning, generally formulated as a graph partitioning problem, is an important step in the physical design of circuits [38]. The connection matrix is one of the storage forms of undirected graphs, so the key to convert a circuit to an undirected graph is to convert the circuit to a connection matrix.

**Definition 3.**  *The connection matrix M = {m_ij_} of a gate-level circuit is defined as follows*:

(3) $$m_{ij}=\{\begin{array}{c} 1,\sigma (v_{i},v_{j})>0\;\&\;i\;\neq \;j\\ 0,\;else \end{array}$$

**Figure 3:**
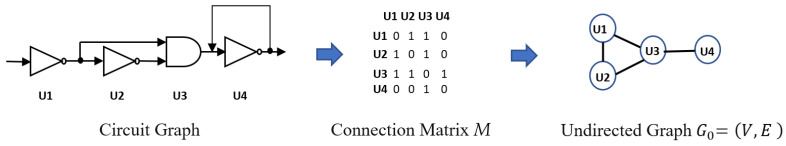
Converting the gate-level circuit to an undirected graph. The circuit contains four logic gates, where U1, U2, and U4 are NOT gates, and U3 is the AND gate. The corresponding connection matrix is *M*. U1 and U4 have no direct connection relationship; thus, the corresponding elements *m*_14_ and *m*_41_ are 0. If there is a connection relationship between other electronic elements, the corresponding element value is 1.

The size of the connection matrix is N*N; *N* is the number of electronic components, each row and column correspond to an electronic component, and $m_{ij}$ represents the connection relationship between electronic components $v_{i}\;\mathrm{and}\;v_{j}$. For ease of partitioning, we remove the self-loop and discrete node situations, unify the electronic components, and use 1 to represent the connection relationship between the electronic components, while 0 represents no connection. A simple example of the gate-level circuit conversion to an undirected graph is shown in Figure 3.

### 3.2. Clustering Algorithm Based on Betweenness Centrality

The clustering algorithm proposed in this paper is mainly used to realize the coarse partition of the original graph. It outputs all the identified clusters *Vc* and the minimum graph $G_{k}$. Different from the traditional random search, we adopt the non-high betweenness maximum degree value $v_{max}$ as the starting node of the BFS algorithm. In addition, the nodes with high betweenness centrality are applied as the search boundary because they play the role of connecting different clusters in the graph. Additionally, the search process ends when one of the following conditions is satisfied: (1) BFS algorithm searches a node belonging to *Y*, and the number of searched nodes is greater than the lower limit *LR*. (2) The number of searched nodes is greater than the upper limit *UR*. 

Finally, all the nodes searched in each round are regarded as cluster $V_{i}$. The above process is repeated until *k* clusters are found; then, the clustering algorithm terminates and outputs a minimum graph. 

The main process of the clustering algorithm based on betweenness centrality is shown in Algorithm 1.
**Algorithm 1:** A clustering algorithm based on betweenness centrality**Input:** $G_{0}$, the number of subsets *k*, high betweenness node set *Y*, lower limit of a cluster *LR*, upper limit *UR*, cluster set *Vc* **Output:** *Vc*, minimum graph $G_{k}$. **Variables:** a cluster $V_{i}$**for** *i* in *k* **do**  $v_{max}\;$= maxdegree_search ($G_{0}\backslash V_{c}$);    //$v_{max}$ does not belong to *Vc*  $V_{i\;}$ = [];  $V_{i}\;$= BFS ($v_{max}$, $G_{0}$, $V_{c}$, $V_{i}$ *Y*, *LR*, *UR*);  $V_{c}$. append ($V_{i}$);             //Store a cluster  **if**  *i* == *k−1*:    $G_{k}$ = $G_{0}$\$V_{c}$;    **return** $G_{k}$;**end for**

Analysis of the following parameters: (1) The lower limit of a cluster *LR* (lower range). If $v_{max}$ node is directly connected to a node in set *Y*, or is particularly close, then BFS would be terminated too early, resulting in the current cluster being too small. Therefore, set a lower limit *LR*. This threshold is a ratio of the number of searched nodes to the total number of nodes, and is dependent on *k.* We set *LR* as (0.3~0.4) in the two-way partition and (0.18~0.28) in the three-way partition. When the nodes in set *Y* are searched, the related BFS algorithm can be terminated if the number of nodes reaches *LR*. In the related experiments in Section 4, we tested all the possible *LR* using the step size 0.02, selecting the best parameter. For example, in the two-way partition, we set *LR* as 0.3, 0.32, 0.34, 0.36, 0.38, and 0.40 in turn, selecting the best *LR*. (2) The upper limit *UR* (upper range). If the starting node $v_{max}$ is particularly far from the nodes in set *Y*, then the BFS algorithm may delay convergence, resulting in the related cluster being too big, which is not conducive to load balancing. Therefore, we set the upper limit *UR* depending on the number of nodes. We set the limit to 0.45 in the two-way partition and 0.3 in the three-way partition. That is, if the number of nodes in $V_{i}$ is greater than *UR*, BFS is terminated. By setting the lower and upper limits of the number of search nodes, the clustering algorithm is helpful for achieving load balancing.

**Figure 4:**
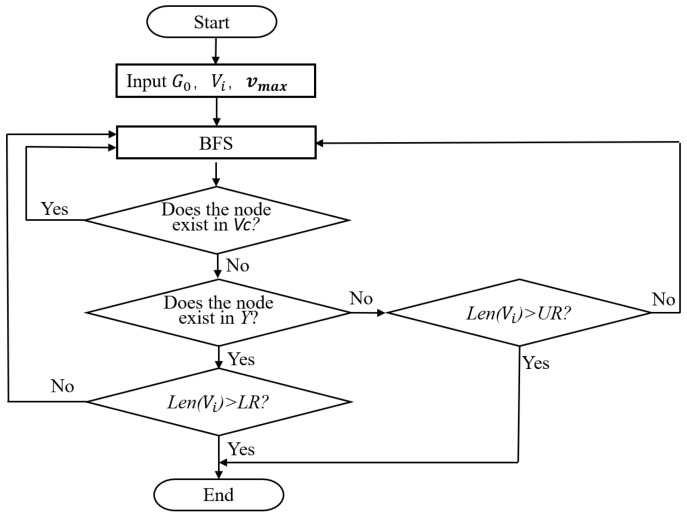
Flowchart of judgment logic.

The related judgment logic is described in Figure 4.

First of all, detect whether the node searched by BFS is in the set *Vc*; if yes, continue the search process; if no, determine whether it is in set *Y*; if yes, determine whether the number of nodes is greater than *LR*; if yes, the algorithm ends; if not, continue the BFS search. If the high betweenness node is not searched, determine whether the number of nodes in $V_{i}$ is greater than *UR*; if it is greater, the algorithm ends, and if it is not greater, continue the BFS search.

The main process of the BFS algorithm with judgment logic is shown in Algorithm 2.
**Algorithm 2**: The BFS algorithm with judgment logic**Input:** $v_{max},$ $G_{0},\;\;V_{c}$, $V_{i}$*LR*, *UR* **Output:** $V_{i}$ **Variable**: The initial value of the queue is an empty list, *stopping condition*: whether the BFS search algorithm has searched for a node that belongs to set *Y*. def BFS ($v_{max}$, $G_{0}$,$\;V_{c}$, $V_{i}$ *Y, LR, UR*):Initialize the queue;while queue:  node = queue.pop(0);  **for** each in node’s all neighbor nodes **do**    **if** each in $V_{c}$:       //Perform the avoidance of duplicate check operation      **continue**;    **if** *stopping condition*:   //Statement determines whether to terminate or not      **if** len($V_{i}$) > *LR*:        **return** $V_{i}$;        **break**;      **elif** each not in $V_{i}$;  //Perform the avoidance of duplicate check operation        the node joins the queue and joins the $V_{i}$;    **else:**
      ** if** len($V_{i}$) > *UR*:        **return** $V_{i}$;        **break**;      **elif** each not in $V_{i}$:        the node joins the queue and joins the $V_{i}$;  **end for**

### 3.3. Constraint-Based Genetic Algorithm

The classic genetic algorithms suffer from two problems when they are applied to circuit partitioning task. First, the convergence speed and final output results would be poor because of the big solution spaces caused by a large size circuit. Second, the probabilistic genetic strategy of traditional genetic algorithms may destroy the clusters in a circuit, thereby negating the result of the coarse partition.

In view of the above two points, we proposed a new genetic strategy using genes on chromosomes. In detail, we define the genes corresponding to the nodes identified by the coarse partition as absolute genetic genes, which do not participate in the crossover and mutation operation and are guaranteed to be inherited into the next generation. On the other hand, the other nodes are treated as traditional probabilistic genetic genes. Because the length of short chromosomes after removing absolute genetic genes is often only about 20–30% of the complete chromosome, the related solution space is only about 5–10% of the traditional area. This is effective for improving the calculation speed and achieving rapid convergence. 

**Figure 5:**
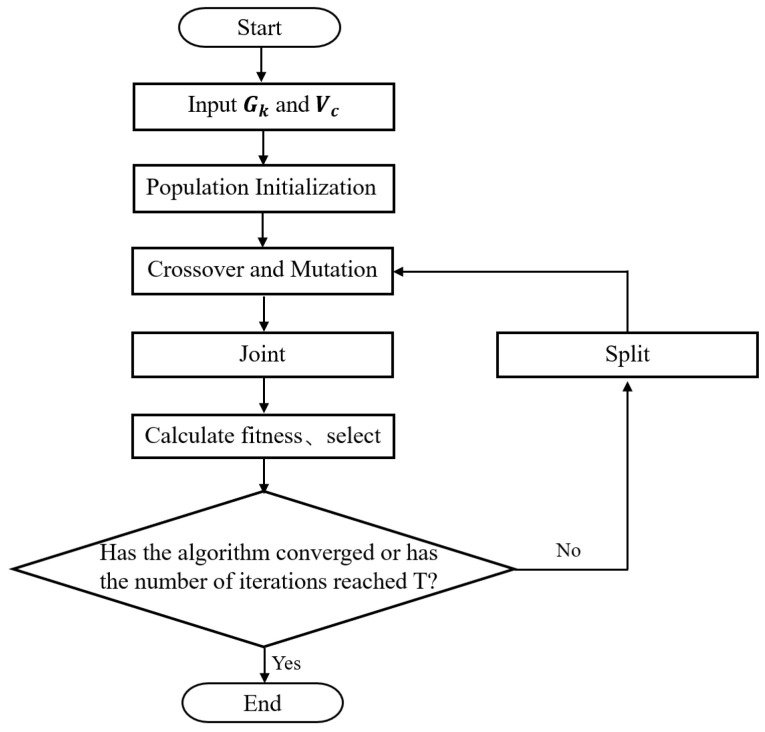
Flowchart of constraint-based genetic algorithm.

Moreover, to ensure that crossover and mutation do not impact the absolute genetic genes, the absolute genetic genes in the complete chromosomes are removed before the next round of evolution, and the remaining genetic information is copied as a new short chromosome to achieve crossover and mutation. This process is realized by the Split function. On the other hand, we also define the Joint function to add absolute genetic genes to a short chromosome to recover a complete chromosome. The flowchart of the entire genetic algorithm is shown in Figure 5.

#### 3.3.1. Chromosome Encoding and Population Initialization

In this paper, a gene represents a node in a graph or an electronic component in a circuit. Each gene has two important parameters: value and subscript. The value of the gene represents which subset the node belongs to, and the subscript corresponds to the mark of the node with the range of values [0, *k* − 1]. For the output of the clustering algorithm, minimum graph $G_{k}$ and cluster set $V_{c}$, there are different encoding rules, as follows:

For the nodes in cluster set $V_{c}$, their related gene values come from their cluster marks. For example, all the nodes of cluster $V_{i}$ have the same gene value *i*. 

**Figure 6:**
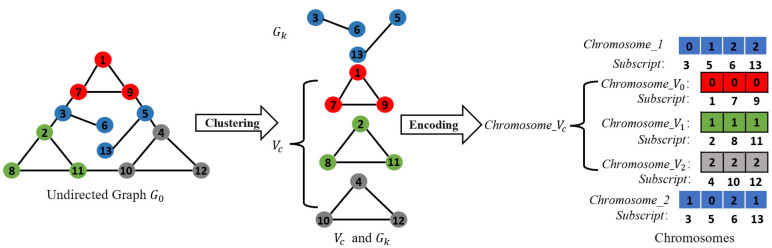
Schematic diagram of chromosome encoding. The nodes of undirected graph *G* are divided into 3 subsets $V_{0}$: {1,7,9},$\;V_{1}$: {2,8,11},$\;V_{2}$: {4,10,12} and minimum graph $G_{k}:$ {3,5,6,13}. The values of all genes on *Chromosome_*$V_{i}$ are the same, 0 on *Chromosome_*$V_{1}$, 1 on *Chromosome_*$V_{2}$ *,* and 2 on *Chromosome_*$V_{2}.$ The values of genes on short chromosomes *Chromosome_1* and *Chromosome_2* in the population are random. The subscripts of all chromosomes in Figure 6 are set according to the marks of their corresponding nodes in *G*.

For the nodes in minimum graph $G_{k}$, they are randomly initialized as *P_size* short chromosomes of population *P*_0_. The length of short chromosomes is $N_{k}$, which is the number of nodes in minimum graph $G_{k}$. The *P_0_* initialization process is as follows: (1) Select each integer in [0, *k* − 1] in turn and add it to a short empty chromosome until the chromosome is full. At this time, the number of each integer in the chromosome is basically the same, about $N_{k}/k$. (2) Shuffle the chromosome to obtain random short chromosomes. (3) Repeat the above operation to obtain *P_size* short chromosomes with a length of $N_{k}$, which is the primary population $P_{0}$. The flowchart of chromosome encoding is shown in Figure 6.

#### 3.3.2. Crossing, Mutation Operators

Crossover: The offspring chromosome first receives all the genes of the father; here, the gene refers to the number [0, *k* − 1]. Then, another chromosome is selected as the mother, randomly generating the crossover point, and the child receives the mother’s gene located at this point. It should be noted that crossovers do not always occur when offspring chromosomes are produced, but they do occur with a certain probability.

Mutation: Each offspring may mutate, and for the k-way partition, the probability that each gene in [0, *k* − 1] mutates into any integer in [0, *k* − 1] (except itself) is the same.

#### 3.3.3. Joint Function

Joint function mainly adds absolute genetic genes to short chromosomes and obtains complete chromosomes for the fitness calculation, as shown in the following equation:

(4) $$V_{0}\cup V_{1}\cup \cdots \cup V_{k-1}\cup V_{k\;}=V_{all}$$

where $V_{c}=\left\{V_{0},V_{1},\cdots ,V_{k-1}\right\}$ means *k* clusters identified by the clustering algorithm. $V_{k}$ contains all the nodes of minimum graph $G_{k}$, that is, the genes of short chromosomes. 

Joint function takes the current population $P_{i}$ as input, and initializes *P_size* empty chromosomes with a length of $N_{all}$ (the number of nodes in $V_{all}$). The subscripts of the chromosomes are arranged from 1 to $N_{all}$, from smallest to the largest, and the assignment of the values of genes on *i*th complete chromosome *CompleteChromosome_i* are as follows:

(1) All genes related to the nodes in $V_{c}$ will be copied to the complete chromosomes, according to subscript. That is, they are absolute genetic genes.
(2) For any chromosome *CompleteChromosome_i*, the other genes are copied from the related short chromosome *Chromosome_i* in the population.

When all values of genes on the *P_size* complete chromosomes are assigned, the new population is outputted.

**Figure 7:**
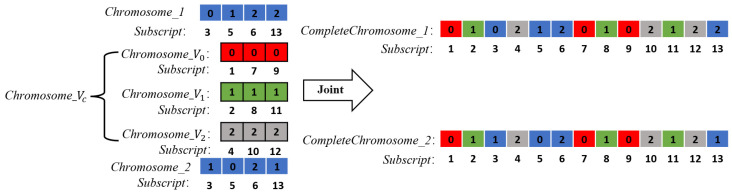
Schematic diagram of the Joint function. *Chromosome_1* and *Chromosome_2* represent two short chromosomes in the current population, *P_size* = 2, and set $V_{0}$, $V_{1}$, and $V_{2}$ are three clusters identified by the clustering algorithm. This function joins the absolute genetic genes with short chromosomes *Chromosome_1* and *Chromosome_2* and outputs two complete chromosomes *CompleteChromosome_1*, *CompleteChromosome_2*.

Taking the three-way partition as an example, the effect of the Joint function is shown in Figure 7.

#### 3.3.4. New Fitness Function

The fitness function is applied to score and evaluate all the chromosomes. Since the coarse partition process has achieved a certain balance, the minimum cutsize is only considered by the fitness function for the fine partition, which is defined as follows:

(5) $$fitness_{i}=C_{max}-C_{i}$$

where $C_{max}$ is the maximum cutsize of chromosomes in the current population, $C_{i}$ is the cutsize of *i*th chromosome, and $fitness_{i}$ is the fitness of the *i*th chromosome.

We use the conventional roulette method to select the optimized chromosome, and the selected probability $per_{i}$ is defined as follows:

(6) $$fitness\_sum=\sum_{i=1}^{P\_size}fitness_{i}$$

(7) $$per_{i}\;=fitness_{i}/fitness\_sum$$

where *P_size* is the size of the population, and *per_i* represents the probability that the *i*th chromosome would be selected. Obviously, Chromosomes with high probability are more likely to be selected, and their genetic factors would gradually expand in the population. This fitness function is beneficial to obtain the fine partition result with the minimum cutsize.

#### 3.3.5. Split Function

To degrade the solution space of the genetic algorithm, before the next round of genetic algorithms begins, it is necessary to perform Split operations on the chromosomes in the population to obtain new short chromosomes and to perform linear transformations on Equation (4) to obtain:

(8) $$V_{k}=V_{all}\backslash V_{0}\backslash V_{1}\backslash \ldots \backslash V_{k-1}$$

According to the above mathematical relationship, the Split function takes the current population as input. The main function is to remove absolute genetic genes from each complete chromosome in the current population.

For the *i*th complete chromosome *CompleteChromosome_i*, all absolute genetic genes in this chromosome are deleted through subscripts stored in $V_{c}$, where $V_{c}=\left\{V_{0},V_{1},\cdots ,V_{k-1}\right\}$. In detail, for any gene in *CompleteChromosome_i*, if its subscript is same as a node mark in $V_{c}$, then it is deleted. The function outputs *P_size* short chromosomes, which is the next generation population, and these are used as input to start the next round of genetic evolution. This operation can reduce the solution space of the overall genetic algorithm and effectively improve the computing efficiency.

**Figure 8:**
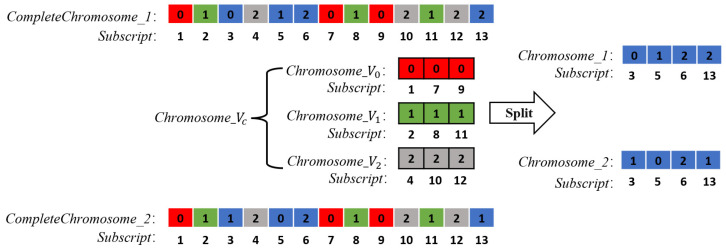
Schematic diagram of Split. The Split function splits the genes contained in $V_{c}$ from the complete chromosome *CompleteChromosome_1*, *CompleteChromosome_2*, then outputs the related short chromosomes *Chromosome_1* and *Chromosome_2*.

Taking the three-way partition as an example, the effect of the Split function is shown in Figure 8.

#### 3.3.6. Constraint-Based Genetic Algorithm

This algorithm takes the number of iterations as the main termination condition, when the number of iterations exceeds the maximum number of iteration *generation_max*, the algorithm terminates. Moreover, when the fitness of all chromosomes in the population is equal, it represents the convergence of the algorithm; at this time, any chromosome in the population is the best partition scheme for the original graph $G_{0}$, and the algorithm is also terminated.

The main process of the constraint-based genetic algorithm is shown in Algorithm 3.
**Algorithm 3:** Constraint-based genetic algorithm**Input:** $G_{0}$, *Vc*
 **Output:** The partition scheme
**Variable:** The current population $P_{i}$, the maximum evolution generation of population *generation_max*, *i* is the generation number, *Crossover_rate*, *Mutation_rate*.
**for** *i* in generation_max **do**  Crossover ($P_{i}$, *Crossover_rate*);  Mutation ($P_{i}$, *Mutation_rate*);  Joint ($P_{i},V_{c}$);  selection ($P_{i}^{j}$);  **if** *stopping condition*:    **return** $P_{i}\left[0\right]$;    **break**;  e**lse**:    $P_{i+1}=$ Split ($P_{i},V_{c})$;**end for**


### 3.4. Complexity Analysis on the Complete Circuit Partitioning Algorithm

For the clustering algorithm, the time complexity of searching the adjacent nodes of a node is O(*n*), and *n* is the number of nodes in a circuit graph. Then, the time complexity of searching all the adjacent nodes is O($n^{2}$).

For the genetic algorithm, there are *P_size* chromosomes in a population; because of the clustering algorithm, there are about 0.2*n genes on each chromosome, the time complexity of gene exchange and mutation is O(0.2*n*), the time complexity of selection is set to O(*n*), the probability of chromosome crossover is p (p ≈ 1), and the probability of mutation is q (0 < q << 1). Then, in the process of inheritance of a generation, the time complexity is O(p* *P_size* *0.2*n* + q* *P_size* + *n*). Suppose the iteration is *b*; thus, the total time complexity is O(b*p*P_size *0.2*n* + b*q* *P_size* + b*n). Considering the fact that P_size *b is always set as O(*n)*, the final time complexity is O($n^{2}$).

Therefore, the complete time complexity of the proposed algorithm is O($n^{2}$).

For space complexity, the clustering algorithm needs to use an auxiliary queue, and in the worst case scenario, all nodes need to enter the queue once, and the space complexity is O(*n*). Additionally, in the worst case scenario, the genetic algorithm requires *P_size* lists with length *n*, so the space complexity is O(P_size*n), and total space complexity is O(P_size*n).

## 4. Experimental Results and Analysis

In order to verify the efficiency of the proposed algorithm, we conducted a series of performance evaluations and comparison experiments. The algorithm is implemented in Python, and all tests are completed on a laptop with a CPU basic frequency of 1.4 GHz, 8 GB memory, and a Windows 10 operating system. There are 12 circuits: (1) Cjtag is the timing conversion circuit of JTAG; (2) Mmu is the memory interface management circuit; (3) Other circuits come from ISCAS ‘89, ISCAS ‘85 standard test cases. In the preprocessing phase, we remove the self-loop, discrete node situation. The number of logic gates and signal lines are listed in Table 1. Moreover, the connection matrix before and after gate-level circuits preprocessing for all test circuits can be found at the link below (the gate-level circuit connection matrix file of the circuit samples can be obtained from https://gitee.com/beacon97/circuit_partition, accessed on 28 March 2023). The experimental goal is to verify the efficiency of the proposed algorithm for finding the minimum cutsize through two sets of experiments: two-way partition and three-way partition.

### 4.1. Analysis of the Results of the Two-Way Partition

The proposed algorithm is compared with the famous partition tool Metis, the classic KL algorithm, and the Gene (traditional gene) algorithm. Among these, Metis was obtained from Karypis Lab [21], and the KL algorithm was obtained from the literature [5]. In order to improve the validity of the experiment, all the above algorithms were carried out 20 times, and the minimum cutsize (Min) and the average cutsize (Avg) were recorded. All experimental results were recorded under the premise of controlling the balance factor β within 0.2. The parameters were set as: *k* = 2, *LR* ∈(0.3~0.4), *UR* = 0.45, *generation_max* = 100, *Crossover_rate* = 0.8, and *Mutation_rate* = 0.003. The cutsize results of the two-way partition experiment on 12 test circuits are shown in Table 2. The smallest Min and Avg in each set of experiments are marked in bold.

**Figure 9:**
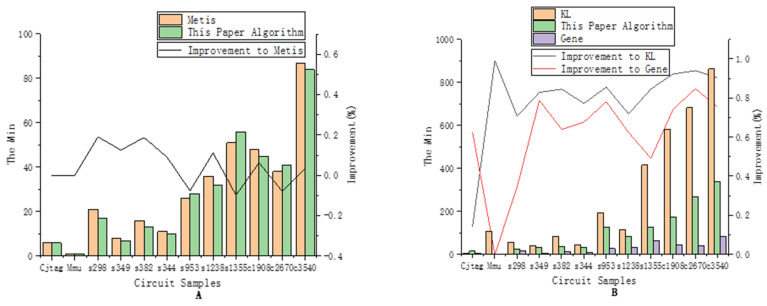
Schematic diagram of two-way partition results. The polyline in (**A**) represents the performance improvement rate of the proposed algorithm compared to Metis, which increases of {0, 0, 19.05%, 12.50%, 18.75%, 9.09%, −7.69%, 11.11%, −9.80%, 6.25%, −7.89%, 3.45%}, respectively. The black polyline in (**B**) represents increases of {14.29%, 99.97%, 70.69%, 82.93%, 84.52%, 77.27%, 85.60%, 71.90%, 84.62%, 92.29%, 93.99%, 90.28%}. The red polyline in Figure B represents increases of {62.50%, 0, 34.62%, 78.79%, 63.89%, 67.74%, 78.13%, 62.35%,49.21%, 74.29%, 84.76%, 75.15%}. The given bar charts illustrate the result of the algorithm applied to the test circuits in terms of the Min.

Figure 9A shows the results of the two-way partition using the proposed algorithm and Metis, and Figure 9B shows the results of the proposed algorithm and the KL and Gene algorithms. The *y*-axis represents the minimum cutsize Min. The *y*-axis on the other side represents improvement.

According to Table 3, the proposed algorithm obtained the best result (Min) 9 times in 12 circuit samples, and the best performance can be improved by up to 19.05%. In Figure 9A, the polyline is generally above the zero-dot line, and is improved by an average of 4.57% compared with Metis. The above experimental results show that compared with the Metis algorithm, the proposed algorithm exhibits a good advantage in finding the Min. On the other hand, the stability of the proposed algorithm (Avg) is weaker than that of Metis, which is related to the random crossover and mutation of the genetic algorithm, so the proposed algorithm must be carried out multiple times to obtain the optimal results.

Compared with the KL and Gene algorithms, the proposed algorithm obtains the best results in all 12 samples. The black and red polylines represent the performance improvement rate of the algorithm compared to KL and Gene, respectively, and the two polylines are both above the zero-dot line. Among the 12 samples, the proposed algorithm improved by an average of 78.95%, compared with the KL algorithm, and 60.95%, compared with the Gene algorithm. From the above experimental results, it is concluded that compared with the KL and Gene algorithms, the proposed algorithm shows great advantages in finding the minimum cutsize Min. The stability of the proposed algorithm is also advantageous over those of the KL and Gene algorithms.

### 4.2. Analysis of the Results of the Three-Way Partition

The KL algorithm only supports two-way partition experiments, so in the three-way partition experiment, only the proposed algorithm, the Gene algorithm, and the Metis algorithm are compared. The parameters are set as: *k* = 3, *LR* ∈(0.18~0.28), *UR* = 0.4, *generation_max* = 100, *Crossover_rate* = 0.8, and *Mutation_rate* = 0.003. The cutsize results of the three-way partition experiments on 12 test circuits are shown in Table 3.

**Figure 10:**
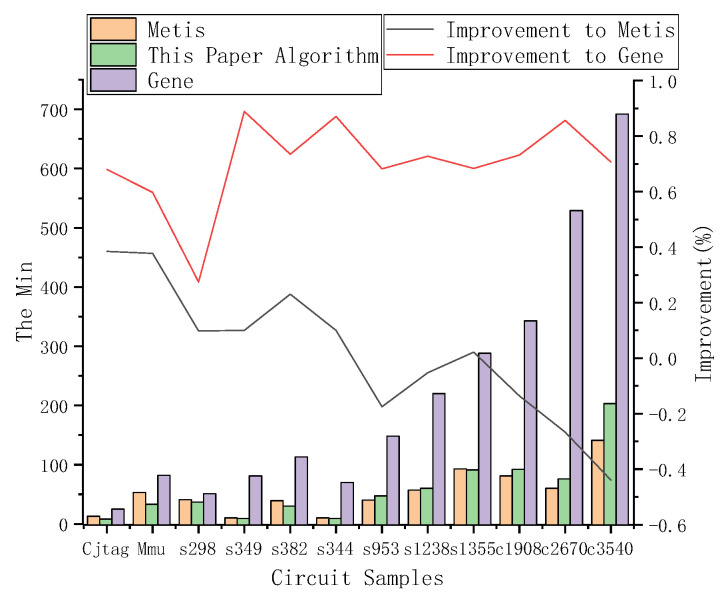
Schematic diagram of three-way partition results. The black polyline in Figure 10 represents the performance improvement rate of the proposed algorithm compared to Metis, which increases by {38.46%, 37.74%, 9.76%, 10.00%, 23.08%, 10.00%, −17.50%, −5.26%, 2.15%, −13.58%, −26.67%, −43.97%}, respectively, and the red polyline in Figure 9 represents the improvement to the Gene algorithm, which is {68.00%, 59.76%, 27.45%, 88.89%, 73.45%, 87.14%, 68.24%, 72.73%, 68.40%, 73.18%, 85.63%, 70.66%}. The bar chart of figure shows the partitioning results of Metis, the Gene algorithm, and the proposed algorithm applied to the 12 circuit samples.

Figure 10 shows the results of the three-way partition of the algorithm compared with the Metis and Gene algorithms in finding the Min.

According to Table 3, compared with the Metis and Gene algorithms, the algorithm proposed in this paper obtains 7 times better results for finding the minimum cutsize Min. In Figure 10, the black polyline is generally above the zero-dot line. Among the 12 circuit samples, the proposed algorithm improved by an average of 2.02% over Metis. The red polyline is also above zero, and the average improvement rate is 70.29%. The above experimental results show that the proposed algorithm shows a good advantage for finding the minimum cutsize in the three-way partition.

## 5. Conclusions

Aiming at the gate-level circuit partitioning problem faced by EDA simulation, we propose a gate-level circuit partitioning algorithm based on clustering and an improved genetic algorithm. By introducing the betweenness centrality, the clustering algorithm is designed to quickly identify clusters in a circuit and realize the coarse partition. In the fine partition phase, a constraint-based genetic algorithm is proposed which realizes a seamless connection with the coarse partition and is effective in obtaining a better partition result. The test results of 12 circuits show that the proposed algorithm exhibits better performance than Metis and traditional genetic algorithms in searching for the minimum number of connections between subsets, which is effective for improving the partition quality.

The algorithm in this paper is relatively insufficient in terms of processing the circuit scale, and the next step will be based on the big data development platform to further improve the overall performance of the algorithm.

## Figures and Tables

**Table 1:** Information table of circuits before and after preprocessing.

Circuit	The Number of Logic Gates before and after Preprocessing	The Number of Signal Lines before and after Preprocessing
Cjtag	68/66	141/140
Mmu	302/236	391/360
s298	142/139	237/237
s349	196/180	227/221
s382	188/180	286/285
s344	195/188	241/241
s953	463/426	729/722
s1238	554/532	565/554
c1355	619/617	1089/1089
c1908	938/936	1519/1519
c2670	1642/1389	2288/2117
c3540	1741/1741	2958/2958

**Table 2:** Cutsize results table in two-way partition.

Circuit	Metis		KL		Gene		Proposed Algorithm	
	Min	Avg	Min	Avg	Min	Avg	Min/*LR*	Avg
Cjtag	6	**6**	7	21	16	25	**6**/0.3	8
Mmu	1	1	108	123	1	18	**1**/0.3	**1**
s298	21	21	58	61	26	43	**17**/0.3	26
s349	8	**8**	41	72	33	62	**7**/0.3	12
s382	16	**16**	84	96	36	41	**13**/0.3	21
s344	11	**11**	44	56	31	97	**10**/0.3	23
s953	**26**	**26**	195	216	128	143	28/0.32	36
s1238	36	36	114	176	85	93	**32**/0.34	43
c1355	**51**	**51**	416	432	126	285	64/0.34	72
c1908	48	**48**	583	634	175	243	**45**/0.34	53
c2670	**38**	**38**	682	704	269	302	41/0.36	47
c3540	87	**87**	864	971	338	376	**84**/0.40	91

**Table 3:** Cutsize results table in the three-way partition.

Circuit	Metis		Gene		Proposed Algorithm	
	Min	Avg	Min	Avg	Min/*LR*	Avg
Cjtag	13	13	25	33	**8**/0.18	**10**
Mmu	53	53	82	86	**33**/0.18	**37**
s298	41	**41**	51	68	**37**/0.18	49
s349	10	**10**	81	92	**9**/0.18	**10**
s382	39	**39**	113	126	**30**/0.18	41
s344	10	**10**	70	87	**9**/0.18	11
s953	**40**	**40**	148	189	47/0.20	51
s1238	**57**	**57**	220	248	60/0.24	65
c1355	93	**93**	288	317	**91**/0.26	96
c1908	**81**	**81**	343	403	92/0.28	102
c2670	**60**	**60**	529	581	76/0.28	79
c3540	**141**	**141**	692	743	203/0.28	224

## Data Availability

The data that support the findings of this study are available on request from the first author (chengrui@csu.edu.cn). Part of data can be obtained from http://gitee.com/beacon97.
